# Supplementary material for: Human Papillary and Reticular Fibroblasts Show Distinct Functions on Tumor Behavior in 3D-Organotypic Cultures Mimicking Melanoma and HNSCC
Source: Int J Mol Sci. 2022 Oct 1;23(19):11651. doi: 10.3390/ijms231911651 (PMC9570214; doi:10.3390/ijms231911651)
Supplement: Supplementary file 1 [file ijms-23-11651-s001.zip › ijms-1916841-supplementary.pdf]

Table S1: Primary antibody specifications

Antibody	Cat.#	Host	Dilution	Source
Collagen type IV	PHM12	Mouse	1:150	Chemicon, Temecula, USA
Ki67	MIB1	Mouse	1:75	Dako, Glostrup, Germany
Keratin 17	CK-E3	Mouse	1:25	Novus Biologicals, Colorado, USA
Keratin 10	DE-K10	Mouse	1:100	Labvision/neomarkers, California, USA
Vimentin	EPR3776	Rabbit	1:1000	Abcam
α-SMA	1A4	Mouse	1:400	Abcam
ZEB2	HPA003456	Rabbit	1:250	Atlas antibodies
ZEB1	HPA027524	Rabbit	1:666	Atlas antibodies
β-catenin	610154	Mouse	1:250	BD Bioscience
SPARC	15274-1-AP	Rabbit	1:400	Proteintech
NKI/beteb	ab34165	Mouse	1:100	Abcam
Pro-COL11A1	CI0011	Mouse	1:125	Oncomatrix

Table S2: qPCR primer specifications

Gene	Forward primer 5’> 3’	Reverse primer 5’> 3’
**Genes of interest**		
SERPINB4	CAAAGGGCAGTGGGAGAATA	CCTCCAGCAAGGCAAAATTA
MMP9	CCTGGAGACCTGAGAACCAA	ATTTCGACTCTCCACGCATC
SNAI2	ACAAGCAGCTGCACTGTGAT	ACACAAGGCAATGTGTGGGT
N-cadherin	TCATTGCCATCCTGCTCTGCAT	AGTTGTTTGGCCTGGCGTTCTT
CLEC3B	CTCAAGAGCCGTCTGGACACC	AAGCATTTCATGTGCACCTTGG
COL7A1	GTACCAGGACCCTGAAGCTC	GTACCAGCGCAGGGTGTAG
MMP12	ATGCAGCTGTTTTTAACCCACG	TGGGATAACCAGGGTCCATCA
MMP3	AGGCTTTCCCAAGCAAATAGC	GGGTCAAACTCCAACTGTGA
COL11A1	AAAGCACTTCGCTTCCTGGG	GACAGTCTTTTCATAGCCTTTTCTG
HAS1	TACTTTTGGGGATGACCGGC	GACGAGGGCGTCTCTGAGTA
ITGβ2	GGGATGGACCGCTACCTCAT	GAATGCCGATCAGCACGATG
MCAM	TCATTGATGGAGTGCCTGTG	CTGTTTCTCCACCTCCAGTAGA
POSTN	TCATTGATGGAGTGCCTGTG	CTGTTTCTCCACCTCCAGTAGA
SPP1	AGAAGTTTCGCAGACCTGACA	GGGATGGCCTTGTATGCACC
VCAM	CAGGCTGGAAGAAGCAGAAAG	TGTCTCCTTCTTTGACACTCTCAG
SOX10	TGAAGGCAGGAAGGAGTTGGC	TGGAGGTTGTAGTGGAGGAGGAC
MMP9	CGACGTCTTCCAGTACCGAG	CTGGTTCAACTCACTCCGGG
SPARC	CTGGACCAGCACCCCATTGA	AGGTCTCGAAAAAGCGGGTG
TGFβ3	CGGGCTTTGGACACCAATTAC	CCCAGATCCTGTCGGAAGTC
ITGα5	CCTGCTCATCCAGAATGGGG	CGATGTGAATCGGCGAGAGT
TMEM132A	AAATCCTGGTGTCTGAGCGG	CTCCAGTCAGTGGTGCTGTA
TMEFF1	TGCCCTGAAAACCTCAATGGT	TTTCACAGTGCTGTCCAGTGT
ZEB2	AAGTACCAGCGGAAACAAGGA	ACAGACAGGAGTCGGAGTCT
SERPINE1	TCCACAAATCAGACGGCAGC	TCGTAGTAATGGCCATCGGG

FZD7	GCTCATGAACAAGTTCGGCTT	GAGCCGTCCGACGTGTTCT
α-SMA	CTCGGGCTGAAGAAGGAACCCAA	ATTGGCACACGGCAGGTGGT
CCRL1	TGAGGGTCCTACAGAGCCAACCA	CTCCCCCTTCCCCCAACCCA
TGM2	GGTGTCCCTGCAGAACCCGC	CGGGGTCTGGGATCTCCACCG
MGP	GCCATCCTGGCCGCCTTAGC	TTGGTCCCTCGGCGCTTCCT
**Reference genes**		
RPS29	TATGTGCCGCCAGTGTTTCC	TGCCCCGGATAATCCTCTGA
ZNF410	GCTGTGGTAAGCAGTTTACTACAG	CTTGGGCTTCACAAAGGAAAGG

Figure S1: The mRNA expression of SOX10 in melanoma FTMs

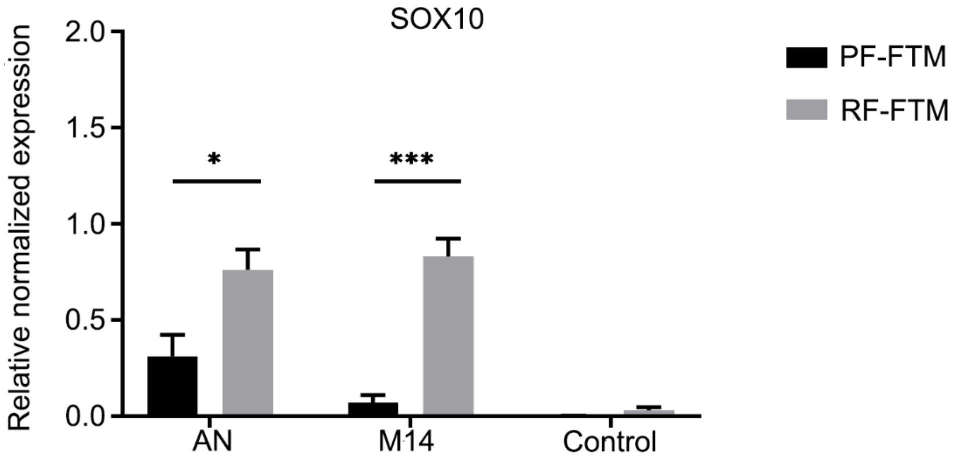

Data was collected from three independent experiments and presented as mean ± standard deviation (SD); * $p < 0.05$, *** $p < 0.001$.

Figure S2: The quantification of NKI-Beteb, K17 and K10 in melanoma and HNSCC FTMs

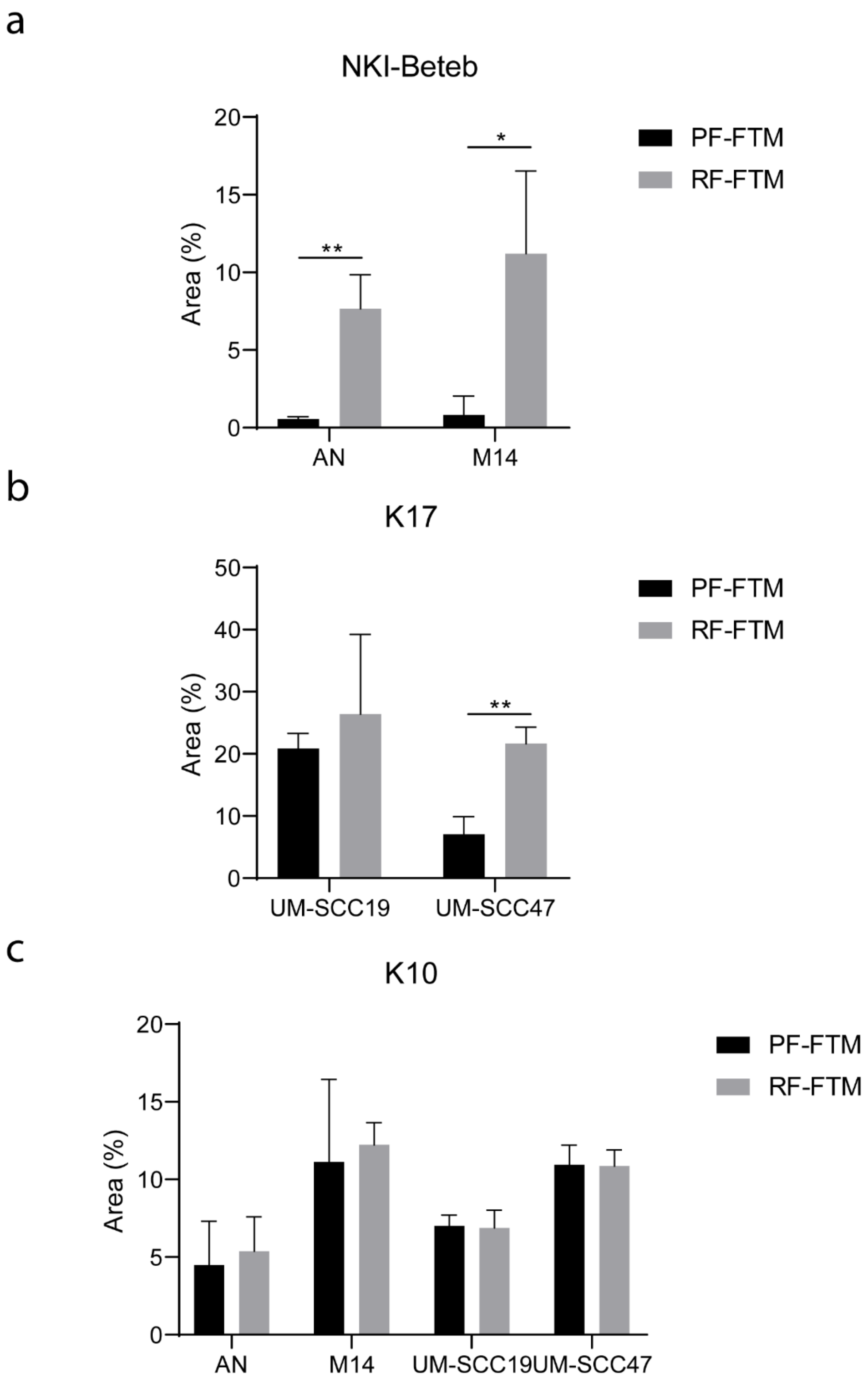

The percentage of positive staining area of (a) NKI-Beteb, (b) K17 and (c) K10 in each FTM was calculated. Data was obtained from three independent experiments and presented as mean ± standard devi-ation (SD); * $p<0.05$; ** $p<0.01$.
